# Implication of Viral Infections for Greenhouse Gas Dynamics in Freshwater Wetlands: Challenges and Perspectives

**DOI:** 10.3389/fmicb.2019.01962

**Published:** 2019-08-27

**Authors:** Giuditta Bonetti, Stacey M. Trevathan-Tackett, Paul E. Carnell, Peter I. Macreadie

**Affiliations:** Faculty of Science, Engineering and Built Environment, School of Life and Environmental Sciences, Centre for Integrative Ecology, Deakin University, Burwood, VIC, Australia

**Keywords:** wetland, freshwater, virus, prokaryote, infection, biogeochemical cycles, carbon dioxide, methane

## Abstract

Viruses are non-living, acellular entities, and the most abundant biological agents on earth. They are widely acknowledged as having the capacity to influence global biogeochemical cycles by infecting the bacterial and archaeal populations that regulate carbon and nutrient turnover. Evidence suggests that the majority of viruses in wetlands are bacteriophages, but despite their importance, studies on how viruses control the prokaryotic community and the concomitant impacts on ecosystem function (such as carbon cycling and greenhouse gas flux) in wetlands are rare. Here we investigate virus-prokaryote interactions in freshwater wetland ecosystems in the context of their potential influence on biogeochemical cycling. Specifically, we (1) synthesize existing literature to establish current understanding of virus-prokaryote interactions, focusing on the implications for wetland greenhouse gas dynamics and (2) identify future research priorities. Viral dynamics in freshwater wetlands have received much less attention compared to those in marine ecosystems. However, based on our literature review, within the last 10 years, viral ecology studies on freshwater wetlands have increased twofold. Despite this increase in literature, the potential implication of viral infections on greenhouse gas emission dynamics is still a knowledge gap. We hypothesize that the rate of greenhouse gas emissions and the pool of sequestered carbon could be strongly linked to the type and rate of viral infection. Viral replication mechanism choice will consequently influence the microbial efficiency of organic matter assimilation and thus the ultimate fate of carbon as a greenhouse gas or stored in soils.

## Introduction

The most widely used definition of wetlands under the Ramsar convention is “areas of marsh, fen, peatland or water, whether natural or artificial, permanent or temporary, with water that is static or flowing, fresh, brackish or salt, including areas of marine water the depth of which at low tide does not exceed six metres.” According to this definition, what is formally known as “wetland” spans a huge and diverse group of ecosystems that are all characterized by the presence of water (temporary or permanently) and aquatic macrophytes (Mitsch and Gosselink, 2007). Although many classification systems have been identified over the past years (Shaw and Fredine, 1956; Cowardin et al., 1979), here we use the classification system adopted by Mitsch and Gosselink (2007), whereby wetlands are classified into seven broad types (see Supplementary Table S1), with a particular focus on freshwater ecosystems. Wetlands are broadly considered one of earth’s most efficient ecosystems for sequestering and storing carbon (Delwart, 2007; Limpens et al., 2008; Dise, 2009; Kirwan and Mudd, 2012), thereby reducing the concentration of atmospheric carbon dioxide (Mitsch and Gosselink, 2007). As governments across the globe try to reduce their greenhouse gas emissions, wetlands have become an important area for both reducing emissions and increasing carbon sequestration. The accurate measurement of the pool of sequestered carbon and gas emissions is crucial to inform overall greenhouse gas budgets, as well as quantifying opportunities for achieving emission reductions through nature-based approaches [“biosequestration”; (Carnell et al., 2018)]. Microbes are often considered the “gatekeepers” of the carbon cycle in marine ecosystems (Arnosti, 2011). In freshwater and coastal wetland ecosystems, the prokaryotic communities are responsible for aerobic (i.e., respiration) and anaerobic (i.e., methanogenesis, denitrification, and sulfate reduction) metabolic pathways that produce greenhouse gases from wetland soils. However, a part of this carbon bypasses microbial remineralization becoming more likely to be permanently sequestered into the soil (Bodelier and Dedysh, 2013; Boon et al., 2014).

In marine and aquatic ecosystems, the “microbial loop” is the pathway that regulates organic matter transfer from prokaryotes to higher trophic levels, but the exact function of prokaryotes in this process is unclear. Different models concerning the existence of the microbial loop in pelagic ecosystems with no benthic influence nor empirical measurements (Azam et al., 1983) and suggesting the role of heterotrophic prokaryotes as carbon sink (Ducklow et al., 1986) or carbon link (Sherr et al., 1987, 1988) have been proposed over the last 40 years. In 1990s, the role viruses were suggested to be key in diverting carbon flow from the higher trophic levels, thereby short-circuiting the microbial loop (Fuhrman, 1999). Viral abundance in soil and aquatic (freshwater and marine) sediments worldwide is estimated to range from 10^7^ to 10^9^ viruses g^−1^ dry mass of sediment (Danovaro et al., 2008a; Williamson et al., 2017). Since viruses need a host to complete their replication cycle, this high abundance of viruses in nature suggests that large numbers of soil-based organisms experience viral infections. In freshwater ecosystems, emerging evidence suggests that bacteriophages likely represent the dominant category of viruses; however, viruses infecting algae, protozoa, and plants might also be presented (Wommack and Colwell, 2000; Jackson and Jackson, 2008).

In this perspective, we focus on the functional role of viruses in freshwater wetlands, discussing the current literature on wetland microbiology and biogeochemical cycling and virus-prokaryote interactions in marine, aquatic, and terrestrial ecosystems. We conclude with three specific questions, followed by our hypotheses, we consider as key points to progress environmental virology in wetlands, focusing on viral infection strategies within the context of carbon cycling and greenhouse gas emission.

### Virus-Prokaryote Interactions

Jackson and Jackson reported in their 2008 review that less than 50 studies have published on topics concerning the keywords “wetland, swamp, pond, bog and virus, phage, temperate, lytic, lysogenic.” The majority of the resulting studies had been conducted in constructed wetlands, rather than natural wetlands. We performed a similar bibliographic research by using the same keywords and search tools (Science Direct, WoK, PubMed, Google Scholar, and Scopus). Only studies with the combination of wetland-related keywords and virus-related keywords in the title, abstract, or keywords were included. Unpublished studies, studies published in languages other than English, and studies on pathogens and pathogen removal were excluded. We found that in the last 10 years since Jackson and Jackson (2008), <30 studies had been published and the research focus on freshwater virology has increased twofold ranging from quantitative and qualitative aspects of the viral ecology to virus-prokaryote interactions, metagenomics, and method development (Supplementary Figures S1A,B, Supplementary Table S1). Most of the studies performed in riparian wetlands (Rooks et al., 2010; Fancello et al., 2013; Kyle and Grant, 2013; Basemer, 2015; Alexyuk et al., 2016; Saxton et al., 2016) and freshwater marshes (Leroy et al., 2008; Thomas et al., 2011; Kyle and Grant, 2013; Zheng et al., 2013; Motlagh et al., 2017; Qin et al., 2017; Dalcin Martins et al., 2018). Few studies were also performed in wetland microcosms (Pradeep Ram and Sime-Ngando, 2008, 2010; Esteban et al., 2015), and few others were in the form of reviews (Middelboe et al., 2008; Wilhelm and Matteson, 2008; Danovaro et al., 2008a; Neori Amir, 2017). The lack of studies in tidal salt marsh and mangrove swamps might be explained with the use of “freshwater” as key word (Supplementary Table S1).

Viruses are widely acknowledged as being the most numerous entity on earth, exceeding prokaryotic abundance by at least one order of magnitude (Danovaro et al., 2008b), yet what is known about the roles of viruses in pelagic ocean ecosystems vastly outweighs our knowledge of viral ecology in wetlands. This limitation is also related to the current challenge of extracting, concentrating, and purifying viral particles from soil material, as well as the complexity of developing and testing several protocols for different soil habitats (Pratama and van Elsas, 2018; Emerson, 2019). The microscale variation within the soil affects the prokaryotic diversity and should then modulate the abundance and diversity of the relative bacteriophages (Fierer et al., 2012; Pratama and van Elsas, 2018). In addition, experiments conducted in thawing permafrost ecosystems suggested that viral infection dynamics might be driven by host lineages (Emerson, 2019). The dynamics underpinning a “top-down” (predation on prokaryotes) or “bottom-up” (resource availability) ecosystem regulation in wetlands remain unclear despite the recognized potential importance of virus-prokaryote interactions in regulating the ocean’s biogeochemical cycles (Pradeep Ram and Sime-Ngando, 2008). Within the microbial loop, protists are bacterivores that have been acknowledged as the major control over the prokaryotes by impacting abundance, production, and community composition. However, the discovery in the late 1980s of the high number of viruses in aquatic ecosystems (10^8^–10^11^) suggested that viruses are also a significant *top-down* control on the prokaryotic populations (Weinbauer, 2004; Fischer et al., 2006; Suttle, 2007) and viral-mediated mortality of prokaryotes makes up ca. 10–50% of the prokaryotic daily production (Mostajir et al., 2015). Since the prokaryotes are an important source of enzymes, which are known to be involved in the carbon cycle (Karner et al., 1994; Bochdansky et al., 1995; Arnosti, 2011), viral-mediated mortality of prokaryotes can stop the carbon from flowing to the upper trophic levels (Pradeep Ram and Sime-Ngando, 2008).

The main focus of viral-relative research to-date has been on their roles as pathogens across a range of ecosystems, rather than their ecological function (Farnell-Jackson and Ward, 2003). As a result of our review, what we do know about viral ecology in marine, aquatic, and terrestrial ecosystems are their role as a major controller of bacterial mortality that results in the diversion of nutrient flows, in preventing algal blooms and promoting genetic exchange across distantly related species as well as their roles as components of sinking particles (Brum et al., 2015; Stroynov and Philippov, 2017; Williamson et al., 2017; Dalcin Martins et al., 2018). Temperate viruses are one of the major causes of mortality for a wide range of organisms, and in the wetlands, it seems that the majority of these viruses are bacteriophages that infect prokaryotes (Wommack and Colwell, 2000; Jackson and Jackson, 2008). The viral infection occurs through a lytic (destruction of the host cell after viral replication) or lysogenic (host cell division with part of the viral genome integrated in the host cell) cycle, and the inclination to one of these replication mechanisms is driven by biotic and/or abiotic factors, such as host availability or the presence of unfavorable conditions for the viral communities (Danovaro et al., 2008a).

The survival of viruses relies upon the number and diversity of their prokaryotic hosts, and in turn, prokaryotic growth and abundance rely on the availability of organic and inorganic matter (Pradeep Ram and Sime-Ngando, 2008). In freshwater environments, the relatively high carbon, nitrogen, and phosphorus availability seems to encourage the choice of a lytic cycle, while the resource limitation seems to yield a lysogenic infection mechanism (Wilson and Mann, 1997; Pradeep Ram and Sime-Ngando, 2010). There is evidence that when phosphorus is limited, the lytic pathway can be hindered due to the resulting negative effect on viral DNA structure (i.e., phosphate backbone; Pradeep Ram and Sime-Ngando, 2010; Thomas et al., 2011).

Acting as *top-down* control, viruses play a key role in regulating the prokaryotic component of the microbial loop. According to the “Kill-the-Winner” model (KtW), by infecting the most abundant population, viruses are predicted to control the food competition among prokaryotic species or functional groups, which is particularly important under resource-limited conditions (Pradeep Ram and Sime-Ngando, 2008). In this *top-down* control scenario, viral lysis releases the prokaryotic dissolved organic carbon (hereafter referred to as “DOC”), which is then recycled back into the system and made available for the local prokaryotic population. In this way, viruses presumably contribute to organic carbon bioavailability and can simultaneously be considered as *bottom-up* controls on the prokaryotic communities that remineralize the “viral-produced” DOC again (Azam et al., 1983; del Giorgio and Williams, 2005; Robinson, 2008). The amount of organic carbon that prokaryotes uptake into their biomass (or synthesis) versus what is respired as CO_2_, CH_4_ or N_2_O depends on the *microbial growth efficiency* (MGE), also referred to as *carbon-use efficiency* (Sterner and Elser, 2002; Manzoni et al., 2012; Wieder et al., 2013).

Knowles and colleagues recently proposed a new model that contrasts the KtW called the “Piggyback the Winner” (PtW) model. The PtW model outlines that during prokaryotic bloom events lysogeny is promoted (low energy cost of superinfection immunity vs. mutation to combat phage infection) resulting in viruses integrating themselves into the host instead of killing it and explaining the low abundance of viruses detected (Knowles et al., 2016; Pratama and van Elsas, 2018). Although the PtW model has been debated taking also into account the evidence of decreasing lysogenic cells with increasing host density observed in aquatic ecosystems (Brum et al., 2016; Weitz et al., 2016), both KtW and PtW models are considered key paradigms to explain virus-host dynamics in soil ecosystems (Pratama and van Elsas, 2018).

From the literature, we know that for prokaryotes in terrestrial soil there is a positive correlation between the MGE and the CO_2_ production, but we lack the mechanistic drivers behind this respiration and carbon assimilation/resynthesis (Schimel, 2013). Based on the literature to-date, we hypothesize that there is an important correlation between the viral infection mechanisms and the MGE that consequently affects on the carbon cycling in wetland soils.

## Wetland Viral Ecology: Questions, Hypotheses, and Future Directions

Based on our literature review and synthesis in the following section, we propose three key questions and our respective hypotheses on virus-prokaryote interactions in freshwater wetlands. These key questions represent the future research directions needed to improve our understanding of the entire microbial loop in freshwater wetlands, and thus, a more complete understanding on how greenhouse gas and carbon sequestration can be managed in these ecosystems.

### How Does Viral Replication Mechanism Influence the Microbial Efficiency, the Carbon Sequestration Rate, and the Greenhouse Gas Emissions in Freshwater Wetlands?

Viruses are acellular and inert entities that require infection of host cells to replicate. Temperate viruses are bacteriophages that accomplish their reproductive cycle *via* a lysogenic or lytic strategy (Ackermann and DuBow, 1987; Weinbauer, 2004). The viral infection through the lytic cycle is a key process in the biogeochemical cycling, especially nutrient turnover, whereby the organic material assimilated by the prokaryotes is released back into the environment once the cell lysis is completed. Additionally, the viral component of the microbial loop is thought to prevent the dominance of few species and niche competition, thus promoting biodiversity (Mostajir et al., 2015). During lytic infection, viruses may also acquire specific functional genes from the host, frequently referred to as auxiliary metabolic genes (AMGs). Recent studies conducted on pelagic marine ecosystems, thawing permafrost, hypolith organisms, and soil viruses have discovered a rich diversity of AMGs capable of altering prokaryotic metabolism and/or improving viral fitness and infection under unfavorable conditions (Hurwitz et al., 2013; Brum and Sullivan, 2015; Pratama and van Elsas, 2018; Sieradzki et al., 2019). These AMGs include genes that encode enzymes involved in functions such as photosynthesis (Mann et al., 2003), metabolism (carbon, nitrogen, sulfur, and lipid; Roux et al., 2016), phosphate regulation (marine; Sullivan et al., 2005), polysaccharide binding, polymer hydrolysis (thawing permafrost; Emerson et al., 2018; Trubl et al., 2018), ribonucleotide reductase (hypolith; Adriaenssens et al., 2015), and atrazine catabolism (soil; Ghosh et al., 2008). Specifically, the virus-encoded polymer hydrolysis might actively contribute to the degradation of complex polymers into smaller forms. The smaller compounds could then be easily accessible to microbes, thus boosting the CO_2_ and CH_4_ production (Emerson et al., 2018; Trubl et al., 2018).

**Figure 1:**
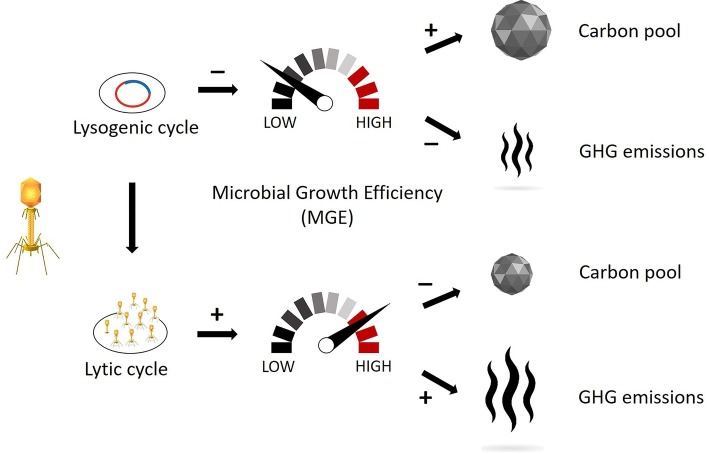
Schematic representation of how different viral replication mechanisms might influence the microbial growth efficiency (MGE) and, consequently, the pool of sequestered carbon and the emissions of greenhouse gas. The lysogenic cycle affects prokaryotic metabolism by decreasing the organic matter usage and thus reducing the carbon decomposition. The lytic cycle affects the carbon turnover by recycling the organic matter already assimilated by prokaryotes and thus increasing the labile carbon available for decomposition. (Vectors used under license from Shutterstock.com).

Viral infections also strongly modulate prokaryotic fitness through infection by the lysogenic cycle, whereby the viral genetic material can be transferred from a lysed to a lysogen host (Horizontal Gene Transfer, HGT). The result would consist of a change in host morphology (lysogenic conversion) and/or the acquisition of immunity from potential phage infection (superinfection immunity; Weinbauer, 2004; Williamson et al., 2017). During lysogeny the viral genome expression may lead to (1) a reduction in the host’s metabolism and (2) a decrease in organic and inorganic matter utilization and growth rate compared to uninfected hosts, as demonstrated with culturable bacteria (Chen et al., 2005; Paul, 2008). Given this, we argue that a strong lysogenic infection might decrease the MGE and the associated substrate usage, resulting in the increase of the carbon not consumed (i.e., remineralized) by the prokaryotic members of the microbial loop. On the other hand, a strong lytic infection and viral shunt might increase the rates of microbial-mediated recycling of the sedimentary organic matter and resulting CO_2_ or CH_4_ production, and we hypothesize that this pathway will have significant negative consequences on carbon cycling and nutrient regeneration in freshwater wetlands, as already demonstrated in marine ecosystems (Corinaldesi et al., 2012). Therefore, we hypothesize that both greenhouse gas emissions and sequestered soil organic carbon are strongly linked to the lytic and lysogenic infection rates (Figure 1), and thus, conditions that trigger one viral strategy over the other may be important levers for manipulation and management of wetland ecosystems.

### How Does Flooding Affect Viral Infection and Organic Matter Dynamics?

As outlined above, viruses play a critical role in diverting the carbon flow from the microbial component back to the ecosystem by short-circuiting the microbial loop. However, in aquatic ecosystems, the presence of viruses within sinking organic aggregates also influences the carbon flux (Weinbauer, 2004; Brum et al., 2015; Guidi et al., 2016). Briefly, it is thought that the DOC released by the viral shunt, i.e., cell lysis, is typically consumed by prokaryotes (Azam et al., 1983; del Giorgio and Williams, 2005; Robinson, 2008). However, recent findings have highlighted how part of the carbon released may coagulate, thus increasing the production rate of aggregates (Weinbauer, 2004; Guidi et al., 2016). On the other side, prokaryotes that colonize sinking particles produce more extracellular enzymes compared to the free-living counterpart and would contribute to aggregate degradation (Weinbauer, 2004). The viral shunt has already been proposed in marine ecosystems as one of the most relevant processes in the production of recalcitrant dissolved organic matter (i.e., “The biological carbon pump”; Jiao et al., 2010). In the marine scenario, the carbon released by the viral shunt was primarily chemically labile (i.e., amenable to assimilation by microbes) but ultimately unavailable for remineralization. Microspatial oxygen depletion (Jiao et al., 2010), protist grazing (Strom et al., 1997), and thermal transformation (Benner and Biddanda, 1998) have been shown to be responsible for making the carbon released by the viral shunt resistant to microbial attack. Similarly, the recalcitrant organic matter formation and sequestration in freshwater wetlands could result from the breakdown of the labile organic matter and the DOC turnover.

In contrast to pelagic marine ecosystems, other scenarios and mechanisms could promote the sequestration of carbon released by the viral shunt. First, the interaction between labile organic matter and mineral soil is recognized as a process to make the labile organic matter inaccessible to the prokaryotes, *via* the formation of recalcitrant soil aggregates or humified organic matter in terrestrial and wetland soils (Davidson and Janssens, 2006; Bridgham et al., 2013a). Second, some wetlands experience regular periods of dryness or drought, e.g., semi-arid wetlands. The resulting reduced water level, soil moisture, and thickness of the water-sediment interface could lead to the reduction in carbon and prokaryotic enzyme diffusion in the porewater, thus reducing the overall metabolic capacity of the soil (Burns and Ryder, 2001). These wetlands starkly contrast to the microbial communities in continually flooded wetlands that do have porewater diffusivity but have to rely on anaerobic metabolic pathways that provide lower energy yields (Davidson and Janssens, 2006).

In dry conditions, soil aggregates produce the spatial heterogeneity that leads to *hot spots* of microbial diversity. It has been recently proposed how the competitive pressure inside these isolated *hot spots* might promote viral infections (Pratama and van Elsas, 2018). For instance, lysogeny has been hypothesized as the dominant infection mechanism in soil and, if shown to occur would emphasize the differences between aquatic and soil viral ecology (Kimura et al., 2008; Emerson, 2019). The potential flooding of the soil can disintegrate the microbial aggregates in the dry soil fostering increased virus-host encounters (Pratama and van Elsas, 2018), thus making wetlands unique wet-dry transitional ecosystems. In arctic freshwater wetlands, it has been observed how the thawing permafrost, and the subsequent transition from a dry to a wet state, was driving the shift from soil-like to aquatic-like viruses, which might carry a higher abundance and diversity of AMGs. In addition, given that the glycoside hydrolyze genes involved in polymer hydrolysis could be transferred between hosts through the horizontal gene transfer, this shift likely suggests potential consequences of dry-wet cycles on the carbon cycle (Emerson et al., 2018; Trubl et al., 2018).

**Figure 2:**
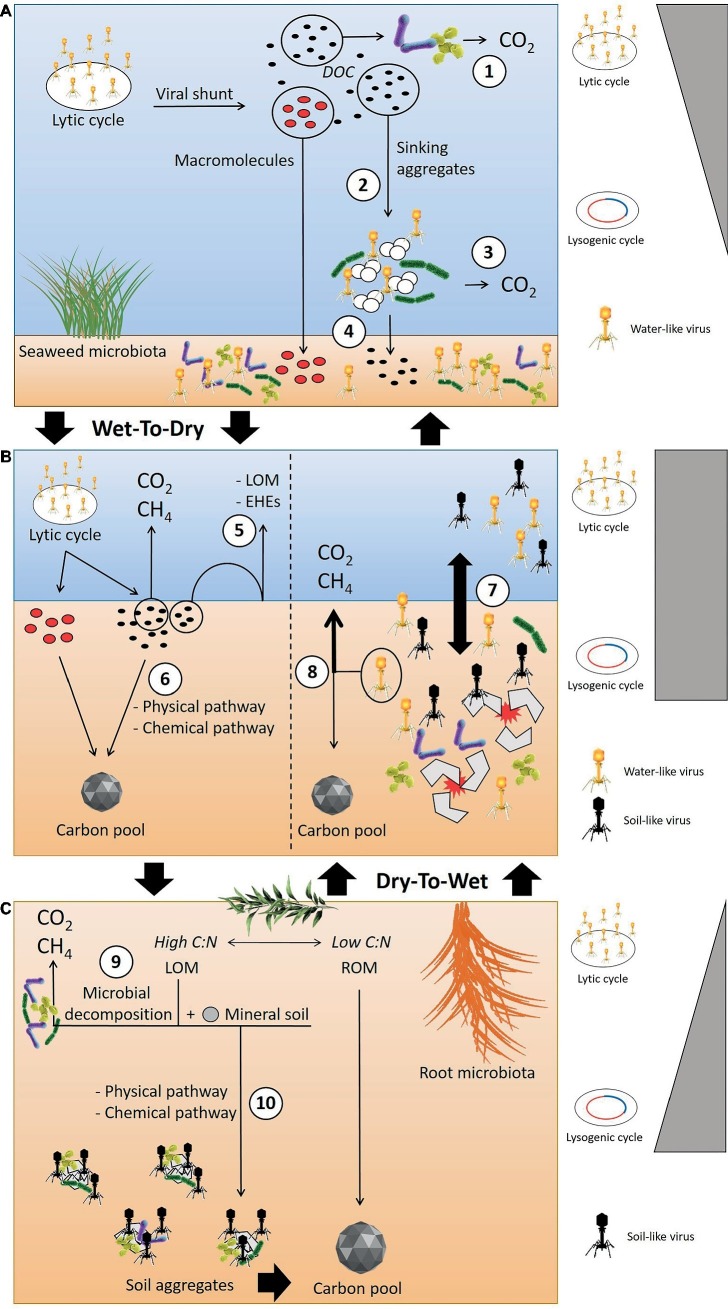
Conceptual model outlining the hypothesized viral infection and organic matter dynamics when in transition from wet-to-dry and dry-to-wet conditions. **(A)** In the water column, the lytic cycle is recognized as the dominant infection strategy. The viral shunt results in the release of DOC that can be directly decomposed by microbes (1) or might coagulate into sinking aggregates (2). However, the high decomposition activities of the microbiota that colonize the sinking aggregates likely degrade the aggregates, leading to the production of GHG (3). The macromolecules released after the viral lysis can be recalcitrant and bypass microbial consumption. These compounds can reach the soil surface, along with labile organic matter escaping microbial attack within the sinking aggregates (4). **(B)** During a wet-to-dry transition, the reduction of the water column and the soil-water interface also reduces the diffusion of labile carbon organic matter (LOM) and extracellular hydrolytic enzymes (EHEs) across the water-soil interface (5), leading to increased remineralization of labile matter within the aerobic layer of the soil. Changes in physiochemical conditions triggered by the occurrence of wet-to-dry cycles can also facilitate the transformation of labile carbon into a stable form through the interaction with mineral soil (6). During dry-to-wet transitions, e.g. flooding events, the breakdown of soil aggregates and presence of the porewater matrix promotes virus-host encounters and the shift from soil-like to water-like viruses (7). This shift could affect the direction of carbon flow, possibly increasing GHG emissions (8). **(C)** In the soil, the fate of LOM can undergo two pathways: (9) microbial decomposition through aerobic or anaerobic reactions, (10) transformation into soil aggregates *via* physical or chemical reactions with mineral soil, i.e., transformation into recalcitrant organic matter (ROM). The promotion of viral infections and lysogeny through aggregate formation has been hypothesized as the dominant infection mechanism in soil. (Vectors used under license from Shutterstock.com).

However, we still lack comprehensive knowledge and understanding of the organic matter and viral infection dynamics in wetlands that undergo wet-dry and/or dry-wet transitions. We hypothesized that the transition from wet to dry climate will trigger physico-chemical conditions, and a portion of the DOC released by the viral lysis might interact with mineral soil and be removed from the microbial attack, thus available for accumulate into the soils (Figure 2). We also hypothesize that in freshwater wetlands characterized by strong flooding events and dry/wet cycles, there is low metabolic or viral activity in the dry phase, but in the transition or flooded phase, the shift from soil-like to water-like viruses will trigger viral infections and the horizontal gene transfer of AMGs directly involved in carbon metabolism will lead to higher greenhouse gas (GHG) emissions (Figure 2).

### How Do Abiotic Factors Affect Virus-Prokaryote Interactions, and Therefore the Carbon Fate, in Freshwater Wetlands?

Alternative to the concept that microbes are the gatekeepers of the carbon cycle, Bridgham et al. (2013b) suggested that microbial communities and their activity are “passive characters” in wetland ecosystems. Instead, the main controls over the biogeochemical cycles are the physiochemical factors, such as dry/wet cycles, hydrology, temperature, and pH. Specifically, shifts in abiotic conditions related to climate change have the potential to enact changes in the microbial members of wetland soils. For instance, a low pH can inhibit the microbial respiration (Benner et al., 1985; Kok et al., 1990; Ye et al., 2012). In Arctic freshwater wetlands, the permafrost thaw progression seems to trigger the GHG production (especially methane) and microbial decomposition rates. By affecting vegetation and hydrology, the permafrost loss causes the increment of nitrogen and labile carbon availability and the transition from hydrogenotrophic to acetoclastic methane production (Hodgkins et al., 2014; McCalley et al., 2014). The virus-host abundance ratios may also be influenced by the increasing thaw and suggest a lineage-specific response to habitat changing (Emerson et al., 2018). In addition, the hydrology in freshwater wetlands can affect the horizontal carbon transport and its dispersion through water flow. In this way, carbon, as well as viral or microbial communities, can be redistributed and accumulated in adjacent systems (Jackson and Jackson, 2008).

Temperature is likely to be a major control in anaerobic biogeochemical processes, such as methanogenesis and denitrification (Gutknecht et al., 2006). In laboratory experiments, it has been demonstrated that microbial sensitivity to temperature might be strongly dependent on the substrate lability (Frey et al., 2013). As we mentioned above, the microbial growth efficiency is a pivotal factor in carbon dynamics, since it determines the journey the carbon will undertake when introduced into the microbial cell. The microbial growth efficiency of relatively more recalcitrant organic matter seems to be inversely correlated to temperature, which may be related to the depletion of labile compounds observed in warmer conditions (Frey et al., 2013). Soil respiration appears to be stimulated by a short-term temperature increases but not by a long-term exposure at higher temperature, suggesting a strong metabolic adaptation that might be explained by the limitation of labile carbon availability (Heimann and Reichstein, 2008; Frey et al., 2013). High soil respiration rates lead to the improvement of the decomposition efficiency that should contribute to an increase in the organic matter remineralization. However, the actual constant organic matter usage with increasing temperature might be justified with a faster carbon consumption and with an increase of new microbial biomass production. This microbial biomass is counterbalanced by the increase of microbial turnover observed at high temperatures and potentially driven by a positive influence of the temperature on grazers (Hagerty et al., 2014) and, possibly, viruses. The positive influence of temperature on viruses might result in high infection rates. The viral lysis of the host cell might cause an initial release of carbon dioxide that added to the carbon dioxide released *via* the prokaryotic respiration pathway, will increase the total amount of greenhouse gas emissions, and will support the infection mechanism hypotheses presented in Question 1.

**Figure 3:**
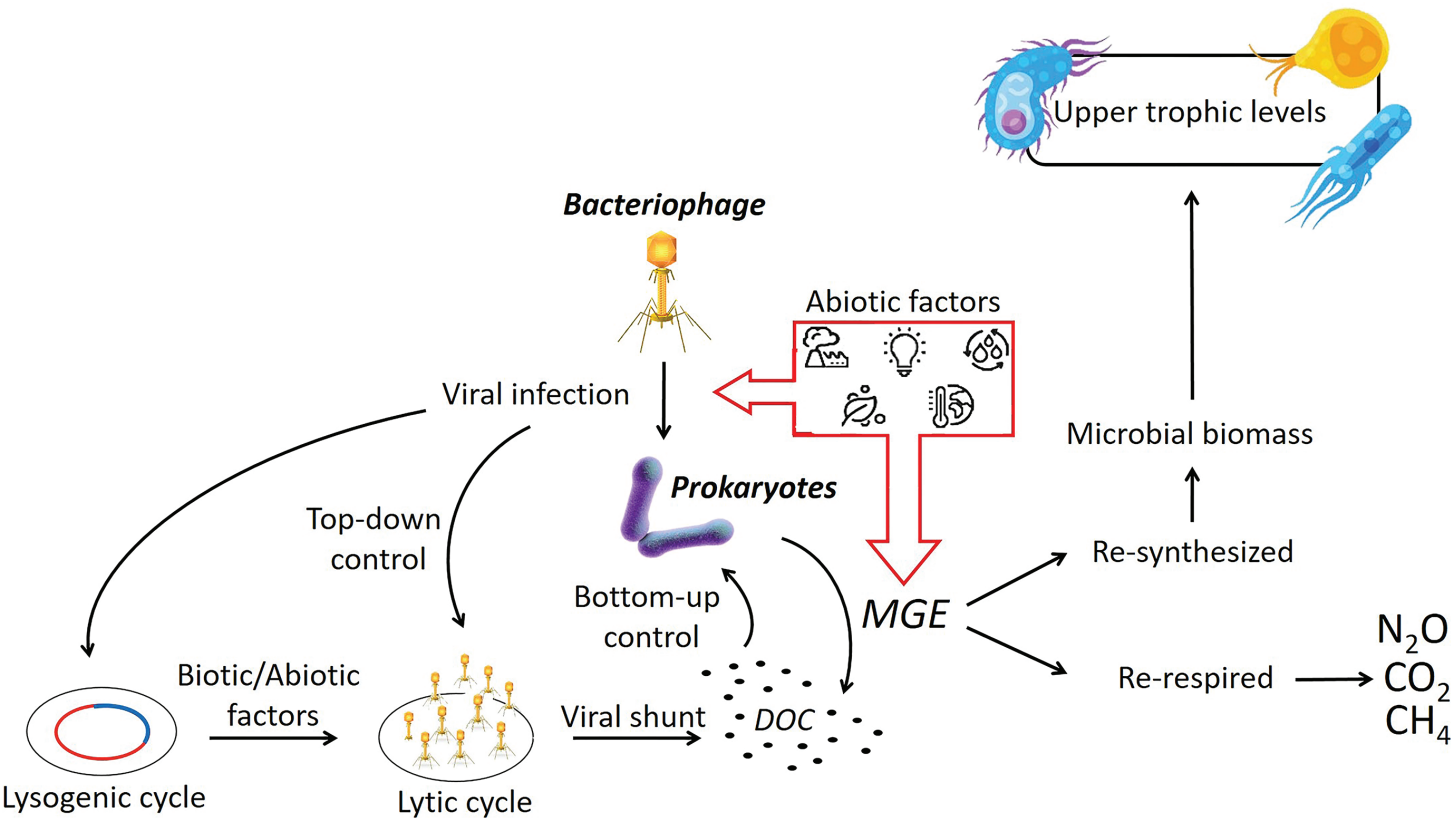
Shifts in abiotic conditions have the potential to enact changes in the viral infection strategy and prokaryotic MGE. The microbial growth efficiency (MGE) defines the proportion of DOC that is being assimilated and then re-synthetized or re-respired with subsequent consequences on the production of carbon emissions. (Vectors used under license from Shutterstock.com).

While we have an understanding of the physiochemical influences on prokaryotes, we know very little about the effects of different abiotic variables on the viral production, infection strategy, or the virus-host relationship in freshwater wetlands, *in vitro* or *in situ* (Figure 3; Middelboe et al., 2008). Some evidence suggests that the viral component might also be a crucial driver in altering the microbial sensitivity to different abiotic factors through the exchange of genetic materials instigated by the unexpected lysis (Boon et al., 2014). In a freshwater microcosm experiment, it has been demonstrated that high levels of nutrients increased the prokaryotic abundance and activity as well as viral production and induced lysogenic cycles by enhancing host cell growth (Mei and Danovaro, 2004; Pradeep Ram and Sime-Ngando, 2008). The effects of temperature changes on viral infection are contradictory, although in general, the lysogenic cycle seems to be induced by temperature increases. On the other hand, stressing the bacterial cell with rapid environmental changes (i.e., temperature, nutrients, and oxygen concentrations) may trigger higher viral replication and the viruses abandoning the host cell before it dies (Boon et al., 2014). In this scenario, failure to complete the lysogenic cycle may cause a reverse to the lytic cycle, leading to increased microbial growth efficiency and thus potentially enhanced carbon utilization.

In light of this evidence, we hypothesize a combined model, whereby the primary factor influencing the virus-prokaryotic dynamic is the abiotic factors actively controlling growth and metabolism, followed by the influence of viral replication mechanisms. A microcosm approach to this question would help to better understand how each abiotic factor separately and collectively influences viral infection and the carbon remineralization activity of infected prokaryotes. Moreover, a controlled approach would allow us to create multiple scenarios that could result in new hypotheses for targets and management strategies to face a changing environment.

## Summary

The fundamental role of prokaryotes in regulating carbon sequestration and greenhouse gas (GHG) emissions in wetlands is widely recognized (Bodelier and Dedysh, 2013). However, wide lens of the identity of the microbes involved, their interactions, potential mortality from viruses and susceptibility to external perturbations (i.e., temperature, wet/dry cycles, and hydrology) and how these components work collectively are still considered a “black box” (Brussaard et al., 2008; Woodcroft et al., 2018). To date, research investigating the microbial communities and functions involved in wetland carbon cycling has primarily focused on the prokaryotic component (bacteria and archaea; Boon et al., 2014). In comparison, the role of viruses has been vastly under-studied, although the ecological virus-prokaryote relationship could be fundamentally to the wetland carbon cycling.

As wetlands are important for biodiversity, ecosystem function, and services, management plans have focused on the restoration of a wetland’s original condition prior to disturbance (e.g., repurposing the land from grazing or cropping and/or changing the hydrology for agricultural or development; Carnell et al., 2018). The restoration of the hydrology and ecosystem functionality has its foundation with the aim of rehabilitating the microbial and biogeochemical functions. If wetland restoration aims to improve carbon sequestration, we propose that management actions will need to address environmental variables that trigger viral infectivity. The factors controlling viral production, viral infection rate, and indeed viral involvement in biogeochemical cycles in these ecosystems will be a key management lever. We also expect that an effective management scenario of freshwater wetlands could include targets for viruses and viral infections based on predicted global change scenarios. While ambitious, the ultimate aim would be to create a functional loop where the positive feedbacks promote carbon remineralization and greenhouse gas emission within the context in the range of abiotic and biotic variables that influence the microbial black box.

## Data Availability

All datasets generated for this study are included in the manuscript and/or the Supplementary Files.

## Author Contributions

GB contributed to manuscript idea and visualization, conducted an investigation process, and wrote the original draft preparation. ST-T, PC, and PM wrote, reviewed, and edited the manuscript and supervised the study.

### Conflict of Interest Statement

The authors declare that the research was conducted in the absence of any commercial or financial relationships that could be construed as a potential conflict of interest.

## Figures and Tables

**Table 1:** 

*Bacteriophage*	Type of viruses infects only prokaryotes (Bacteria and Archaea) as hosts.
*Burst size*	Number of viral particles released with the lysis of a single-prokaryotic cell. This number depends on the viral replication rate inside the host.
*Dissolved organic carbon (DOC)*	The fraction of total organic carbon dissolved into marine- and freshwater. The size of particles ranges between 0.7 and 0.22 μm.
*Immobilization*	Process by which the mineralized matter is converted into organic matter by retention as microbial biomass.
*Labile organic matter*	Organic matter that represents the major bioavailable source of food for microbes. This organic matter is characterized by a high C/N ratio and a short resident time within the soil.
*Lysogenic cycle*	One of the two cycles of bacteriophage reproduction. It results in the prokaryotic cell division with part of the viral genome integrated in the host genome, which is called prophage.
*Lytic cycle*	One of the two cycles of bacteriophage reproduction. It results in the destruction of the host after the viral replication.
*Mineralization*	Aerobic and anaerobic oxidative process by which the labile organic remains are converted into mineral compounds (inorganic forms or less complex organic forms).
*Microbial growth efficiency (MGE)*	Total amount of organic carbon that prokaryotes assimilate.
*Microbial loop*	Pathway that regulates the DOC transfer from prokaryotes to higher trophic levels as microbial biomass.
*Recalcitrant organic matter*	Organic matter resistant to microbial attack and, therefore, to microbial decomposition. This organic matter is characterized by a low C/N ratio or has physical-chemical protection that leads to a long resident time within the soil.
*Temperate phage*	Bacteriophage that replicates through both lytic and lysogenic cycle.
*Viral shunt*	Viral breakdown of the prokaryotic cell. Essential source of organic detritus contributes highly to the metabolism of other prokaryotes.
