# Supplementary material for: Implication of Viral Infections for Greenhouse Gas Dynamics in Freshwater Wetlands: Challenges and Perspectives
Source: Front Microbiol. 2019 Aug 27;10:1962. doi: 10.3389/fmicb.2019.01962 (PMC6718870; doi:10.3389/fmicb.2019.01962)
Supplement: Supplementary file 1 [file Data_Sheet_1.docx]

**SUPPLEMENTARY MATERIAL**

**Title:** Implication of viral infections for greenhouse gas dynamics in freshwater wetlands: challenges and perspectives

**Authors:** Giuditta Bonetti ^a,*^, Stacey M. Trevathan-Tackett^a^, Paul Carnell ^a^, Peter I. Macreadie ^a^

^a^ School of Life & Environmental Sciences, Centre for Integrative Ecology, Deakin University, 221 Burwood Hwy, Burwood VIC, 3125, Australia


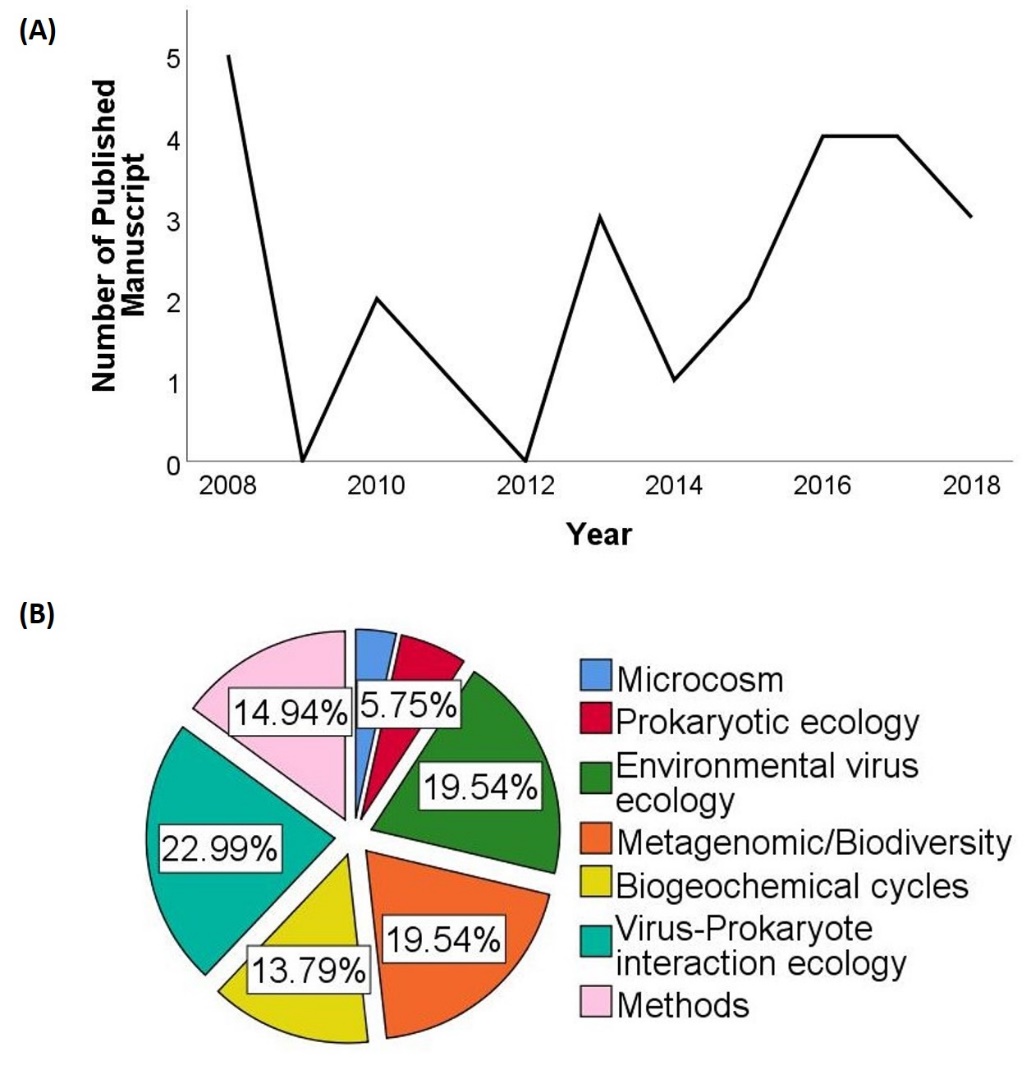


**Figure 1S.** Graphic summary of studies that have examined the viruses in wetland ecosystems in the last 10 years. The online scientific database search occurred in December 2018 on (A) Number of studies per year; (B) Viral topics explored expressed as percentage.

**Table S1.** Numbers and reference of studies that have examined virus and virus-prokaryote interactions organized into seven broad types following Mitsch & Gosselink (2007), with wetland microcosms and reviews as additional categories. Data are provided according to our bibliographic research from online scientific databases in December 2018.

| **Wetland type** | **Number of studies** | **References** |
| --- | --- | --- |
| Tidal salt marsh | 0 |  |
| Tidal freshwater marsh | 0 |  |
| Mangrove swamps | 0 |  |
| Freshwater marsh | 7 | (1–7) |
| Peatlands, bogs and fens | 2 | (8–12) |
| Forested wetlands | 1 | (13) |
| Riparian wetlands | 6 | (6,14–18) |
| Wetland microcosms | 3 | (19–21) |
| Reviews | 4 | (22–25) |

**References**

1. Leroy M, Prigent M, Dutertre M, Confalonieri F, Dubow M. Bacteriophage morphotype and genome diversity in Seine River sediment. Freshw Biol. 2008;53(6):1176–85.

2. Dalcin Martins P, Danczak RE, Roux S, Frank J, Borton MA, Wolfe RA, et al. Viral and metabolic controls on high rates of microbial sulfur and carbon cycling in wetland ecosystems. Microbiome. 2018;6(1):138.

3. Qin K, Ji X, Zhang C, Ding Y, Kuang A, Zhang S, et al. Isolation and characterization of wetland VSW-3, a novel lytic cold-active bacteriophage of Pseudomonas fluorescens. Can J Microbiol. 2017;63(2):110–8.

4. Thomas R, Berdjeb L, Sime-Ngando T, Jacquet S. Viral abundance, production, decay rates and life strategies (lysogeny versus lysis) in Lake Bourget (France). Environ Microbiol. 2011;13(3):616–30.

5. Zheng C, Wang G, Liu J, Song C, Gao H, Liu X. Characterization of the Major Capsid Genes (g23) of T4-Type Bacteriophages in the Wetlands of Northeast China. Microb Ecol. 2013;65(3):616–25.

6. Jennifer E. Kyle & F. Grant. Geochemistry of Virus–Prokaryote Interactions in Freshwater and Acid Mine Drainage Environments, Ontario, Canada. Geomicrobiol J. 2013;30, 769–78.

7. Mohaghegh Motlagh A, Bhattacharjee AS, Coutinho FH, Dutilh BE CS and GR. Insights of Phage-Host Interaction in Hypersaline Ecosystem through Metagenomics Analyses. Front Microbiol. 2017;8:352.

8. Ballaud F, Dufresne A, Francez A-J, Colombet J S-NT and QA. Dynamics of Viral Abundance and Diversity in a Sphagnum-Dominated Peatland: Temporal Fluctuations Prevail Over Habitat. Front Microbiol. 2016;6:1494.

9. Stroynov Ya.V. PAD. Bacterio- and virioplankton in water bodies of a raised bog (Vologda oblast, Russia). Inl Water Biol. 2017;10(1):37–43.

10. Emerson JB, Roux S, Brum JR, Bolduc B, Woodcroft BJ, Jang H Bin, et al. Host-linked soil viral ecology along a permafrost thaw gradient. Nat Microbiol [Internet]. 2018;3(8):870–80. Available from: http://dx.doi.org/10.1038/s41564-018-0190-y

11. Trubl G, Solonenko N, Chittick L, Solonenko SA, Rich VI, Sullivan MB. Optimization of viral resuspension methods for carbon-rich soils along a permafrost thaw gradient. PeerJ. 2016;4(C):e1999.

12. Trubl G, Jang H Bin, Roux S, Emerson JB, Solonenko N, Vik DR, et al. Soil Viruses Are Underexplored Players in Ecosystem Carbon Processing. mSystems. 2018;3(5):1–21.

13. Williamson KE, Fuhrmann JJ, Wommack KE, Radosevich M. Viruses in Soil Ecosystems: An Unknown Quantity Within an Unexplored Territory. Annu Rev Virol. 2017;4(1):201–19.

14. Alexyuk, Madina Saparbaevna et al. “Diversity of viral photosystem-II psb A genes in the largest channel reservoir of Kazakhstan.” Res J Pharm Biol Chem Sci. 2016;7.3:2674–2680.

15. Saxton MA, Naqvi NS, Rahman F, Thompson CP, Chambers RM, Kaste JM WK. Site-specific environmental factors control bacterial and viral diversity in stormwater retention ponds. Aquat Microb Ecol. 2016;77:23-36.

16. Rooks DJ, Smith DL, Mcdonald JE, Woodward MJ, Mccarthy AJ, Allison HE. 454-pyrosequencing: A molecular battiscope for freshwater viral ecology. Genes (Basel). 2010;1(2):210–26.

17. Fancello L, Trape S, Robert C, Boyer M, Popgeorgiev N, Raoult D, et al. Viruses in the desert: A metagenomic survey of viral communities in four perennial ponds of the Mauritanian Sahara. ISME J. 2013;7(2):359–69.

18. Basemer K. Biodiversity, community structure and function of biofilms in stream ecosystems. Res Microbiol. 2015;166(10):774–81.

19. Esteban DJ, Hysa B, Bartow-McKenney C. Temporal and spatial distribution of the microbial community of winogradsky columns. PLoS One. 2015;10(8):1–21.

20. Sime-Ngando ASPR and T. Functional responses of prokaryotes and viruses to grazer effects and nutrient additions in freshwater microcosms. ISME J. 2008;2, 498–509.

21. Pradeep Ram AS, Sime-Ngando T. Resources drive trade-off between viral lifestyles in the plankton: Evidence from freshwater microbial microcosms. Environ Microbiol. 2010;12(2):467–79.

22. Midelboe, M. , Jacquet, S. and Weinebauer M. Viruses in freshwater ecosystems: an introduction to the exploration of viruses in new aquatic habitats. Freshw Biol. 2008;53: 1069-1075.

23. Neori Amir AM. The Functioning of Rhizosphere Biota in Wetlands – a Review. Wetlands. 2017;37(4):615–33.

24. Wilhelm, S. W. and Matteson AR. Freshwater and marine virioplankton: a brief overview of commonalities and differences. Freshw Biol. 2008;53: 1076-1089.

25. Danovaro R, Corinaldesi C, Filippini M, Fischer UR, Gessner MO, Jacquet S, et al. Viriobenthos in freshwater and marine sediments: A review. Freshw Biol. 2008;53(6):1186–213.
